# Expression of full-length dystrophin reverses muscular dystrophy defects in young and old *mdx*^4cv^ mice

**DOI:** 10.1172/JCI189075

**Published:** 2025-06-10

**Authors:** Hichem Tasfaout, Timothy S. McMillen, Theodore R. Reyes, Christine L. Halbert, Rong Tian, Michael Regnier, Jeffrey S. Chamberlain

**Affiliations:** 1 Department of Neurology and; 2 Senator Paul D. Wellstone Muscular Dystrophy Specialized Research Center, University of Washington School of Medicine, Seattle, Washington, USA.; 3 Department of Bioengineering, College of Engineering and School of Medicine, University of Washington, Seattle, Washington, USA.; 4 Department of Anesthesiology and Pain Medicine, University of Washington School of Medicine, Seattle, Washington, USA.; 5 Mitochondria and Metabolism Center and; 6 Center for Translational Muscle Research, University of Washington, Seattle, Washington, USA.; 7 Department of Biochemistry, University of Washington School of Medicine, Seattle, Washington, USA.

**Keywords:** Genetics, Muscle biology, Therapeutics, Gene therapy, Genetic diseases, Skeletal muscle

## Abstract

Gene replacement therapies mediated by adeno-associated viral (AAV) vectors represent a promising approach for treating genetic diseases. However, their modest packaging capacity (~4.7 kb) remains an important constraint and significantly limits their application for genetic disorders involving large genes. A prominent example is Duchenne muscular dystrophy (DMD), whose protein product dystrophin is generated from a 11.2 kb segment of the *DMD* mRNA. Here, we explored methods that enable efficient expression of full-length dystrophin via triple AAV codelivery. This method exploits the protein *trans*-splicing mechanism mediated by split inteins. We identified a combination of efficient and specific split intein pairs that enabled the reconstitution of full-length dystrophin from 3 dystrophin fragments. We show that systemic delivery of low doses of the myotropic AAVMYO1 in *mdx*^4cv^ mice led to efficient expression of full-length dystrophin in the hind limb, diaphragm, and heart muscles. Notably, muscle morphology and physiology were significantly improved in triple-AAV–treated *mdx*^4cv^ mice versus saline-treated controls. This method shows the feasibility of expressing large proteins from several fragments that were delivered using low doses of myotropic AAV vectors. It can be adapted to other large genes involved in disorders for which gene replacement remains challenged by the modest AAV cargo capacity.

## Abstract 2

This study describes a novel gene therapy method to express large and challenging proteins involved in genetic disorders.

## Graphical abstract

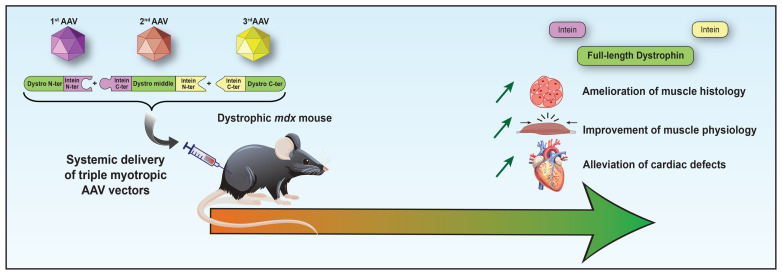

## Introduction

Among various viral and nonviral gene delivery methods, adeno-associated virus (AAV) vectors are the most efficient vehicle to target different organs, such as the liver, brain, and striated muscles (1, 2). Hundreds of natural serotypes have been isolated, and an increasing number of capsids with tissue-specific tropism have been successfully engineered via directed evolution, capsid shuffling, and rational or computer-guided design (3, 4). Although these new capsids dramatically improved specific tissue targeting, allowing the use of lower doses, the relatively small size of AAV capsids (~20 nm) represents a major constraint to their wide application, specifically for indications in which the transgene size exceeds the AAV cargo capacity (~4.7 kb).

Duchenne muscular dystrophy (DMD) is an X-linked disease that results from mutations in the 2.2 MB *DMD* gene that prevent the expression of a functional dystrophin protein (5, 6). Gene replacement of dystrophin using AAVs represents an attractive therapeutic approach. However, the full-length dystrophin (muscle isoform Dp427) coding sequence is approximately 11.2 kb, requiring 3 AAV vectors to carry the full coding region plus gene-regulatory elements. Because of this constraint, several groups have focused instead on developing truncated versions, also known as micro-dystrophin (μDys) (7–12). These miniaturized proteins are stably expressed from AAVs and retain partial dystrophin activity. However, recent preclinical and clinical data suggested incomplete protection of different μDys constructs when administrated to DMD animal models or patients with DMD (13–15). This indicates the necessity of expressing larger constructs with higher functionality.

Several other therapeutic strategies have been developed to express nearly full-length dystrophin, such as exon skipping or gene editing (16–20). These strategies showed promising dystrophin protein expression, but the low efficacy and subtherapeutic levels of dystrophin expressed in striated muscles failed to completely rescue muscle defects. Also, such methods can only be applied to the minority of mutations that disrupt small and nonessential portions of the gene. Similarly, extremely low expression levels of full-length dystrophin were detected following triple AAV infusion combined with *trans*-splicing or homologous recombination between the vector genomes (21, 22). Therefore, successful expression of therapeutic levels of full-length dystrophin remains elusive.

We have previously reported a novel method that utilizes protein *trans*-splicing (PTS) mediated by split inteins to join multiple dystrophin fragments and express a large midi-dystrophin (midi-Dys ΔSR5-15) or the full-length dystrophin (muscle isoform Dp427) (23). PTS is a unique posttranslational reaction described originally in various unicellular micro-organisms, in which 2 halves of a protein are joined into a functional unit by forming a covalent bond mediated by split inteins (24). Several split inteins have been isolated, purified, and studied in vitro and have become powerful tools in protein bioengineering (25). In addition, split inteins have lately been tested in gene replacement strategies to express large and challenging proteins that exceed the AAV cargo capacity (26–28).

Importantly, we identified several split intein pairs with high orthogonality and showed that 1 combination yielded high full-length dystrophin protein levels in vitro using cell culture, or in vivo following intramuscular injections of AAV serotype 6 into *mdx*^4cv^ mice (23). Moreover, systemic delivery of high doses (total dose, 2 × 10^14^ vg/kg) of triple AAV6 resulted in robust expression of full-length dystrophin in hind limb muscles and hearts. However, several clinical reports suggest that these doses might induce severe adverse events such as liver damage and immune reactions, which could limit the viability of this triple vector approach (29, 30).

Here, we report robust expression of full-length dystrophin in hind limb, diaphragm, and heart muscles following the administration of 2 low doses of the potent myotropic AAVMYO1. We show that full-length dystrophin expression restored hind limb muscle force and cardiac defects in young or old *mdx*^4cv^ mice to WT levels. Our results support the feasibility of effective reconstitution of full-length dystrophin from multiple fragments deliverable by low doses of myotropic AAVs.

## Results

### Efficient body-wide expression of dystrophin following systemic triple AAV infusion.

We previously reported the feasibility of inducing expression of a large midi-Dys (ΔSR5-15) and full-length dystrophin following dual or triple AAV infusion in *mdx*^4cv^ mice (23). Using a split GFP system, we screened several intein pairs and identified several sequences with high PTS activity and orthogonality. We characterized 2 combinations: split : inosine-5′-monophosphate dehydrogenase (IMPDH-1) and split gp41.1 or split ribonucleoside-diphosphate reductase (Nrdj1) and split gp41.1. In both cases, gp41.1 was used to join the middle to the C-terminal fragment, while IMPDH-1 and Nrdj1 were assessed to join the N-terminal to the middle fragment. Our data showed that the combination of Nrdj1 and gp41.1 resulted in higher levels of full-length dystrophin expression in vitro using plasmid transfections or in vivo following intramuscular injections of triple AAVs.

**Figure 1:**
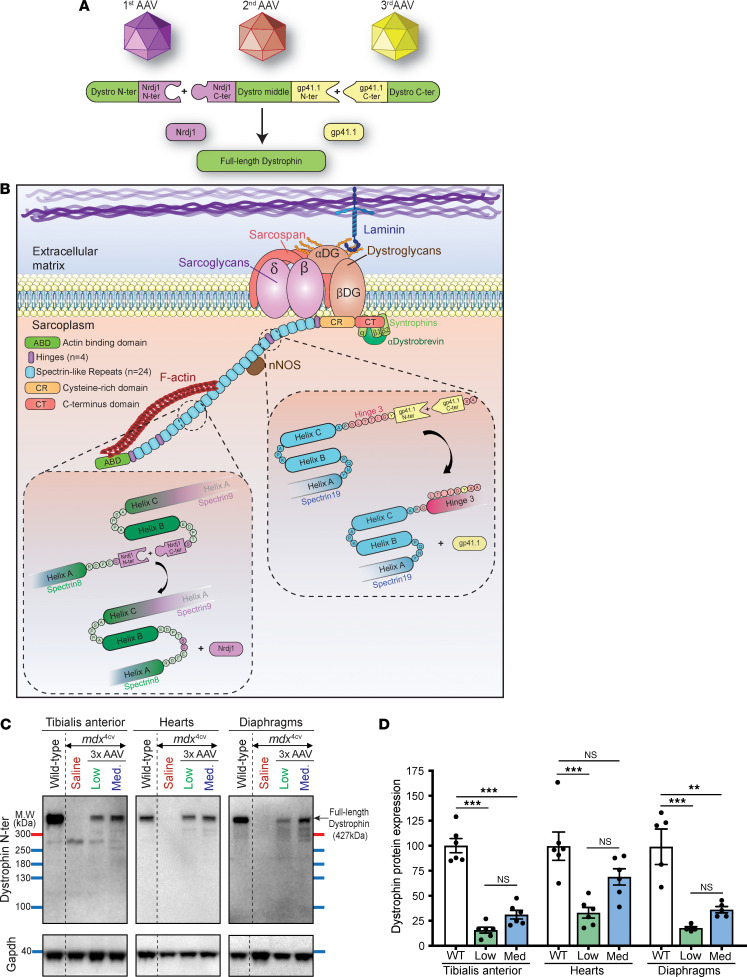
Efficient expression of full-length dystrophin in young *mdx*^4cv^ mice. (**A**) Cartoon depicting the triple AAV approach to express full-length dystrophin via PTS mediated by 2 split inteins. (**B**) Dystrophin structure with the split site used to insert split inteins. (**C**) Western blots showing full-length dystrophin expression in TA, diaphragm, and heart muscle lysates collected from WT mice and young *mdx^Acv^* mice treated with saline- or triple-AAV (3x AAV). (**D**) Dystrophin protein expression normalized to GAPDH and expressed as a percentage versus WT levels. *n* = 5–6 samples per group. Data represent the mean ± SEM. **P < 0.01 and ***P < 0.001, by 1-way ANOVA with Tukey’s post hoc test. Low, low dose (4 × 10^13^ vg/kg); Med, medium dose (8 × 10^13^ vg/kg).

**Figure 2:**
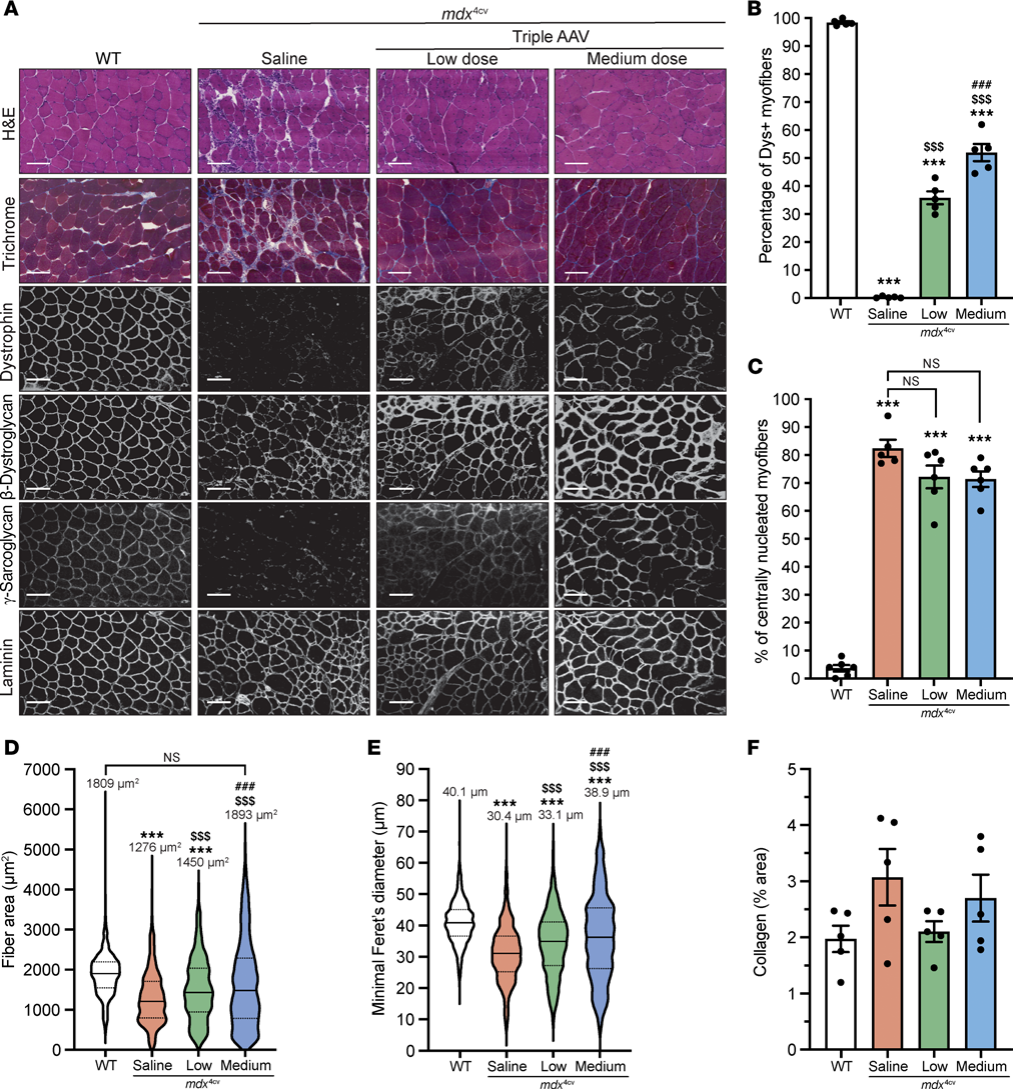
Improvement of TA muscle histology with triple AAV delivery. (**A**) TA cross-sections stained with H&E or Trichrome (top row, scale bars: 100 μm) or immunolabeled for dystrophin (N-terminal antibody), β-dystroglycan, γ-sarcoglycan, or laminin (lower panels, scale bars: 100 μm). Originally captured in RGB colors, image sections were inverted to white color to better visualize the staining. The original panel is presented in Supplemental Figure 1. (**B**) Percentage of dystrophin^+^ (Dys^+^) fibers. *n* = 5 samples per group; *n* = 600–1,000 myofibers counted per sample. (**C**) Percentage of myofibers with central nuclei. *n* = 5–6 samples per group; *n* = 400–700 myofibers analyzed per sample. (**D**) Myofiber area and (**E**) minimal Feret’s diameter measured on TA cross-sections. *n* = 5 samples per group; *n* >700 myofibers analyzed per sample. The average values are shown on top of the violin bars. The solid line represents the median, while the dashed lines show the quartiles. (**F**) The collagen area of TA muscle sections was measured using trichrome-stained cross-sections (no significance was found between the groups). *n* = 5 samples per group. ***P < 0.001 versus WT; ^$$$^*P* < 0.001 versus the saline-treated group; ^###^*P* < 0.001 versus the low-dose-treated group. The mean of each group was compared by 1-way ANOVA with Tukey’s post hoc test. Data represent the mean ± SEM.

Here, we packaged the split dystrophin/intein (Nrdj1/gp41.1 combination, Figure 1, A and B) into AAVMYO1 and evaluated expression levels in 8-week-old *mdx*^4cv^ mice treated systemically with total doses of 4 × 10^13^ vg/kg (low) or 8 × 10^13^ vg/kg (medium) at an equimolar ratio of all 3 vectors [1:1:1]. Three months later, we detected robust expression of full-length dystrophin in protein lysates extracted from the tibialis anterior (TA), heart, and diaphragm (Figure 1, C and D). With the low dose, dystrophin levels were quantified between 20% and 50% versus WT, whereas the medium dose resulted in 2-fold higher expression levels. In TA muscles, dystrophin expression was detected in approximately 38% and approximately 55% of myofibers with, respectively, low and medium doses (Figure 2, A and B). The expression of full-length dystrophin led to a slight reduction of the percentage of centrally nucleated myofibers but restored the expression of β-dystroglycan and γ-sarcoglycan at the myofiber periphery (Figure 2, A and C, and Supplemental Figure 1). We noted significant morphological changes with both AAV doses. Analysis of muscle cross-sections stained with H&E revealed improvements in general muscle histology with very densely packed and larger myofibers and reduced mononuclear cell infiltration. Muscles from *mdx*^4cv^ mice treated with the low dose showed a significant improvement in myofiber area and diameter, but greater amelioration was found with the medium dose with values comparable to those for WT (Figure 2, D and E). At this stage, TA muscle sections stained with trichrome did not show an increase in fibrosis (Figure 2, A and F).

**Figure 3:**
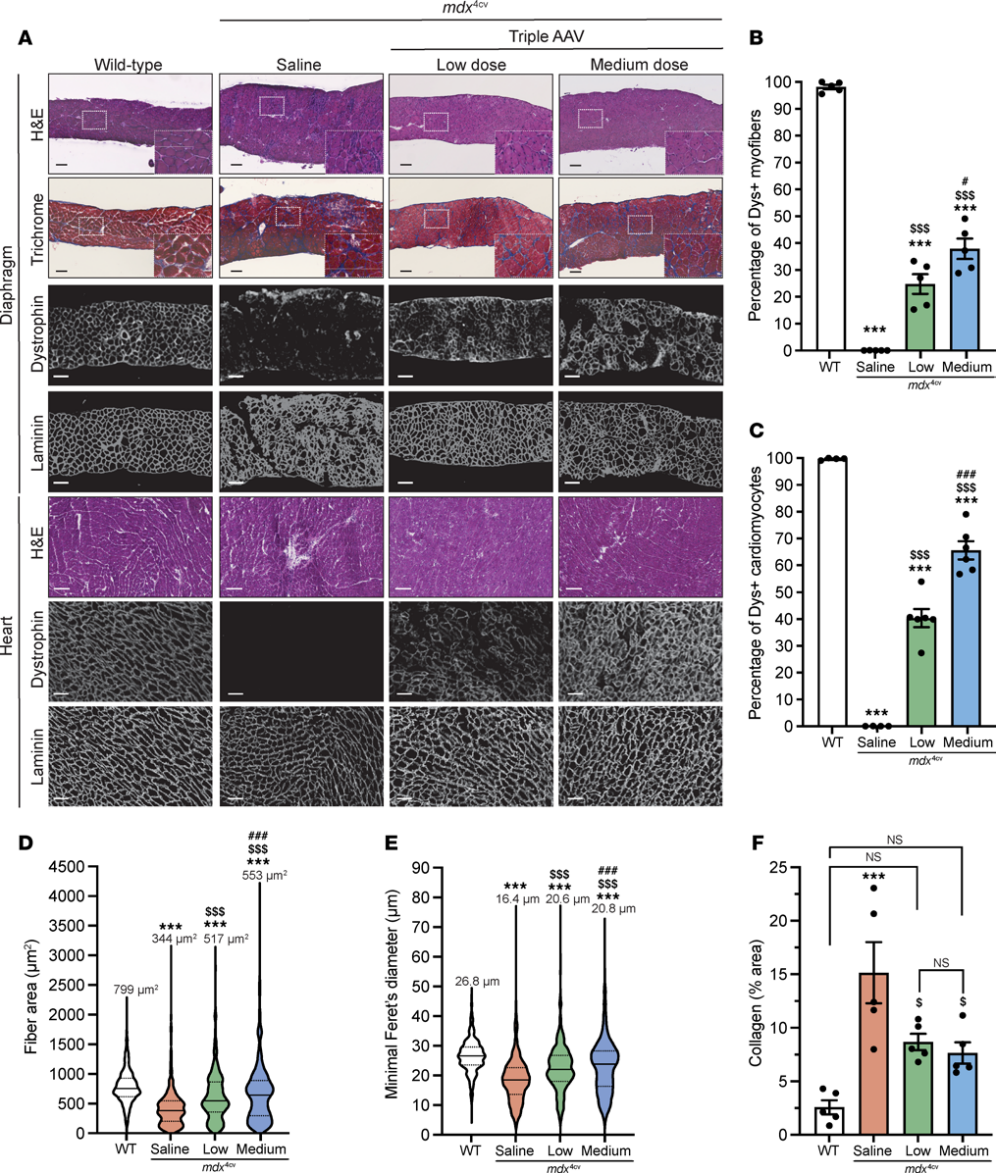
Broad biodistribution of dystrophin expression detected in diaphragm and heart. (**A**) Diaphragm and heart cross-sections stained with H&E or trichrome (scale bars: 100 μm), or immunolabeled for dystrophin (N-terminal antibody) or laminin (scale bars: 100 μm for diaphragms, 50 μm for hearts). The original panel (green and red colors) is presented in Supplemental Figure 2. (**B**) Percentage of dystrophin^+^ fibers quantified on diaphragm sections. *n* = 5 samples per group; *n* = 600–1,000 myofibers counted per sample. (**C**) Percentage of dystrophin^+^ cardiomyocytes. *n* = 5 samples per group; *n* = 600–1,000 cells counted per sample. (**D**) Myofiber area and (**E**) minimal Feret’s diameter measured on diaphragm cross-sections. *n* = 5 samples per group; *n* >300 myofibers analyzed per sample. The average values are shown on top of the violin bars. The solid line represents the median, and the dashed lines show the quartiles. (**F**) The collagen area of the diaphragm samples was measured using cross-sections stained with trichrome. ***P < 0.001 versus WT; ^$^*P* < 0.05 and ^$$$^*P* < 0.001 versus the saline group; ^#^*P* < 0.0 and ^###^*P* < 0.001 versus the low-dose group. The mean of each group was compared by 1-way ANOVA with Tukey’s post hoc test. Data represent the mean ± SEM.

Similarly, we detected dystrophin in the heart and diaphragm sections, with a lower distribution noticed in the diaphragm samples (Figure 3, A–C, and Supplemental Figure 2). With the low dose, approximately 28% of diaphragm myofibers were dystrophin^+^ versus approximately 40% with the medium dose, whereas a higher number of cardiomyocytes showed positive dystrophin staining culminating at approximately 40% and approximately 68% with low and medium doses, respectively (Figure 3, A–C, and Supplemental Figure 2). This expression greatly improved the general diaphragm histology with a larger myofiber area and diameter compared with the saline-treated group, but none of the tested doses restored these 2 parameters to WT levels (Figure 3, D and E). In contrast, we quantified lower collagen content in diaphragm samples with both doses (Figure 3F).

These data showed the feasibility of codelivering 3 AAV vectors to striated muscles and successful expression of full-length dystrophin from ligation of 3 moieties via PTS mediated by 2 split inteins.

### Expression of full-length dystrophin corrects functional defects.

**Figure 4:**
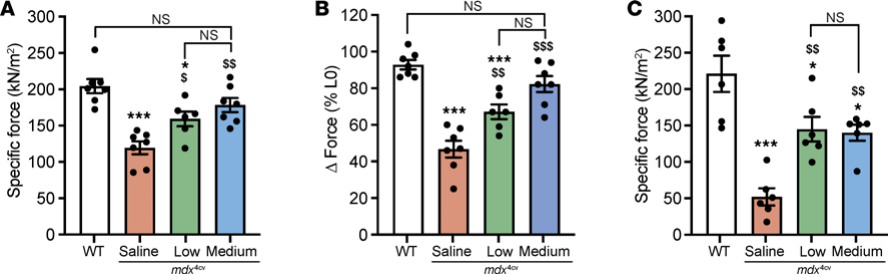
Improvement of TA and diaphragm contractile properties. (**A**) TA muscle–specific force development measured in situ following sciatic nerve stimulation. *n* = 6–7 mice per group. (**B**) Muscle force drop measured following a mechanical injury induced by 20% stretching beyond the optimal L0. *n* = 6–7 mice per group. (**C**) Specific force development measured in vitro using diaphragm strips from *mdx*^4cv^ mice treated with saline or triple AAVMYO1 or from age-matched WT mice. *n* = 5–7 mice per group. Data represent the mean ± SEM. *P < 0.05 and ***P < 0.001 versus WT; ^$^*P* < 0.05, ^$$^*P* < 0.01, and ^$$$^*P* < 0.001 versus the saline-treated group. One-way ANOVA with Tukey’s post hoc test.

To evaluate the phenotypic benefits of the neo-expressed full-length dystrophin for muscle physiology, we assessed specific muscle force and resistance to contraction-induced injury in TA and diaphragm muscles. While saline-treated *mdx*^4cv^ mice exhibited a deficit in TA muscle force and a higher susceptibility to eccentric contraction–induced muscle injury, mice treated with the triple AAV approach showed normalization of TA muscle–specific force development to WT levels and significant protection from muscle damage (Figure 4, A and B, and Supplemental Table 1). The medium dose resulted in slightly higher muscle force levels and better muscle protection. Moreover, diaphragm muscles from the saline-treated group showed dramatic muscle weakness (Figure 4C and Supplemental Table 2). Although lower dystrophin levels were detected in diaphragm samples compared with TA and heart samples, these levels were sufficient to significantly correct the muscle force deficit.

Together, these data demonstrate that the successful reconstitution of full-length dystrophin led to significant improvements in muscle function and morphology in young dystrophic mice treated with low or medium doses of triple AAVMYO1. Additionally, the data suggest that the expression of full-length dystrophin corrected both mechanical defects and energetic deficits, as revealed by the short rest period between stimulation cycles.

### Triple AAV delivery improves skeletal muscle defects in old mdx^4cv^ mice.

**Figure 5:**
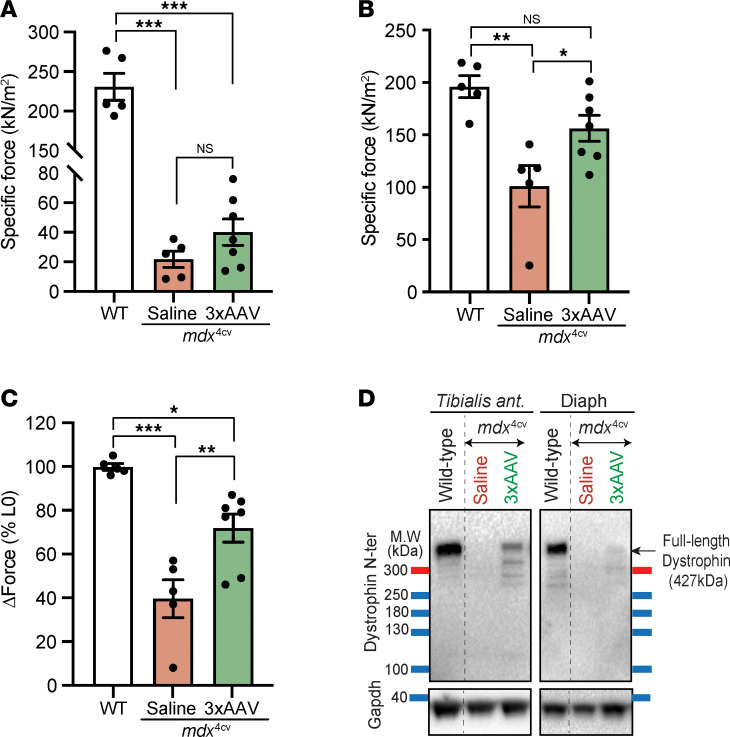
Expression of full-length dystrophin restores TA muscle defects in old and severely affected *mdx*^4cv^ mice. (**A**) Diaphragm specific force recorded in vitro using isolated muscle trips. *n* = 5–6 mice per group. (**B**) Maximal specific force developed by TA muscles at L0 following sciatic nerve stimulation. *n* = 5–7 mice per group. (**C**) Muscle force measured after eccentric contraction injury induced by 15% lengthening beyond L0. *n* = 5–7 mice per group. Data represent the mean ± SEM. *P < 0.05, **P < 0.01, and ***P < 0.001, by 1-way ANOVA with Tukey’s post hoc test. Data represent the mean ± SEM. (**D**) Western blots showing full-length dystrophin expression in TA muscle lysates collected from WT mice and old *mdx*^4cv^ mice treated with saline- or triple AAV, but lower levels were detected in diaphragm samples of the same animals.

As the efficient and broad expression of full-length dystrophin found in the young cohort resulted in dramatic functional changes when a low dose of AAVMYO1 was administrated, we sought to evaluate this approach in severely affected and old *mdx*^4cv^ mice. Low doses (4 × 10^13^ vg/kg total) of triple AAVMYO1 were systemically administrated to 17-month-old male mice. At this age, *mdx*^4cv^ mice were reported to display a severe dystrophic phenotype with deterioration of the diaphragm and cardiac function and pronounced fibrosis and infiltration seen in other skeletal muscles (31, 32). Seven months after triple AAV injection, slight changes were detected in the diaphragm muscle force development in mice treated with split intein/dystrophin vector versus the saline-treated group (Figure 5A); however, the group treated with triple vector AAVs showed significant improvements in TA muscle–specific force to values comparable to WT levels and higher resistance to muscle injury induced by mechanical stretch (Figure 5, B and C). These functional changes reflected the degree of successful reconstitution of full-length dystrophin. We detected low levels of full-length dystrophin in diaphragm protein lysates by Western blot analysis, whereas higher levels were found in TA samples (Figure 5D).

**Figure 6:**
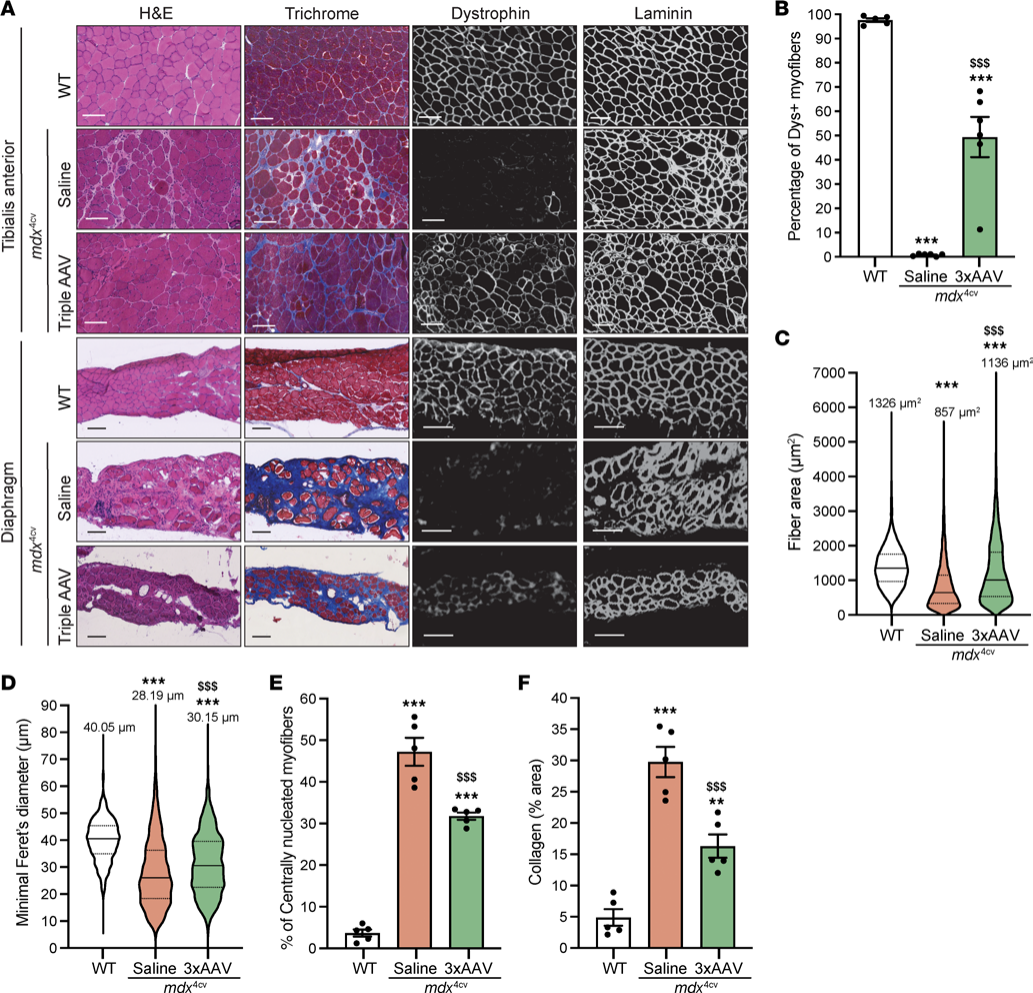
Partial correction of TA muscle histology with triple AAV treatment. (**A**) General muscle morphology of TA and diaphragm muscle cross-sections stained with H&E or trichrome (right columns, scale bars: 100 μm). Dystrophin expression is shown across muscle sections immunolabeled with antibodies against dystrophin or laminin (left columns, scale bars: 100 μm). The original staining (green and red) is displayed in Supplemental Figure 3. (**B**) Percentage of dystrophin^+^ fibers quantified on TA sections. *n* = 5–6 samples per group; *n* = ~2,000 myofibers counted per sample. (**C**) Myofiber area and (**D**) minimal Feret’s diameter determined from TA cross-sections. *n* = 5 samples per group; *n* = 1,000 myofibers analyzed per sample. The average values are shown on top of the violin bars. The solid line represents the median, and the dashed lines show the quartiles. (**E**) Percentage of centrally nucleated myofibers quantified using TA sections stained with H&E. (**F**) The fibrosis area was measured using TA cross-sections stained with trichrome. **P < 0.01 and ***P < 0.001 versus WT; ^$$$^*P* < 0.001 versus the saline group, by 1-way ANOVA with Tukey’s post hoc test. Data represent the mean ± SEM.

These functional data were also in accordance with the general muscle histology for these 2 muscles. While diaphragm muscle sections showed highly fibrotic and infiltrated histology with greatly reduced myofiber content in *mdx*^4cv^ mice treated with saline or triple AAVs, we observed clear improvements in TA muscle sections in the group treated with the triple vector approach (Figure 6A). Moreover, double immunolabeling of these cross-sections using anti-laminin and anti-dystrophin antibodies confirmed the presence of fewer myofibers in diaphragm samples, which were also positive for dystrophin staining. In contrast, immunostaining of TA muscle sections revealed that approximately 60% of myofibers were dystrophin^+^ (Figure 6B and Supplemental Figure 3). In addition, TA muscle cross-sections stained with H&E displayed reduced mononuclear cell infiltration, larger myofibers with a higher cross-sectional area and diameter, and a lower number of centrally nucleated myofibers (Figure 6, A and C–E). Importantly, using trichrome staining, TA cross-sections exhibited reduced collagen deposits that were 20% lower than in the saline-treated group (Figure 6, A and F).

### Normalization of cardiac defects with the triple vector approach.

**Figure 7:**
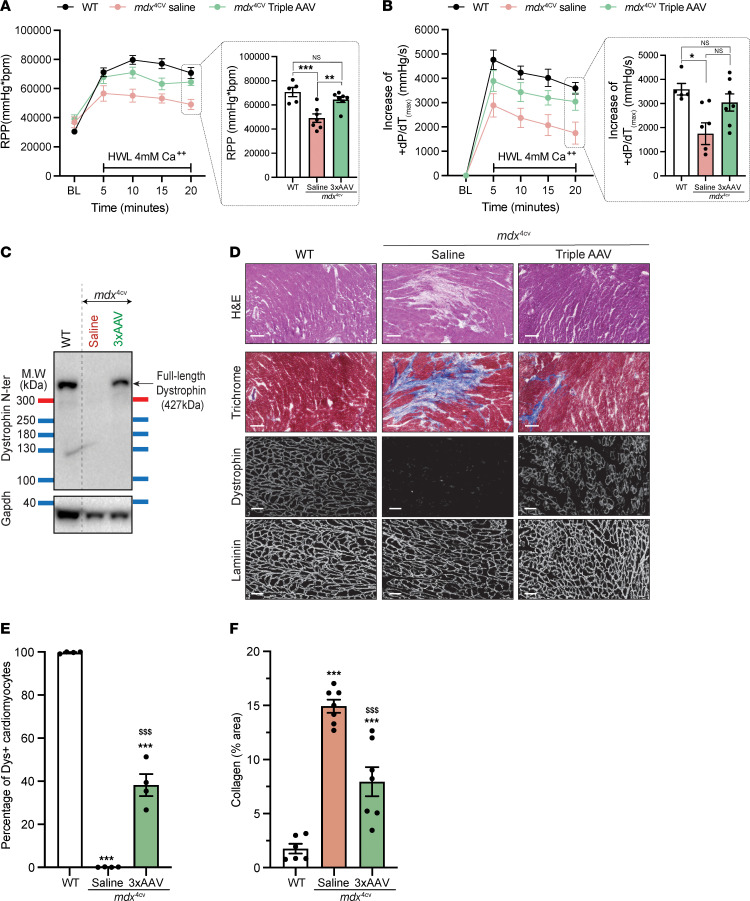
High levels of full-length dystrophin rescue *mdx*^4cv^cardiac defects at the late stage of the disease. (**A**) Rate pressure product (RPP) at baseline (BL) or at different time points under high-workload (HWL) conditions. (**B**) Increase of +dP/dT_(max)_ during high-workload challenge compared with baseline. Data in **A** and **B** represent the mean ± SEM. (**C**) Protein expression in heart tissue analyzed by Western blotting. (**D**) General morphology of the heart tissue assessed with H&E and trichrome staining (top rows, scale bars: 200 μm). Dystrophin expression and distribution were determined by immunolabeling using anti-dystrophin and anti-laminin antibodies (bottom rows, scale bar: 50 μm). (**E**) The percentage of cardiomyocytes expressing dystrophin was determined by dystrophin immunostaining. (**F**) The collagen percentage present in heart sections was quantified using trichrome staining. *P < 0.05, **P < 0.01, and ***P < 0.001 versus WT; ^$$$^*P* < 0.001 versus the saline-treated group. One-way ANOVA with Tukey’s post hoc test. Data represent the mean ± SEM.

The most common cause of death for patients with DMD is cardiac failure due to the lack of dystrophin in cardiomyocytes (33, 34). This cardiac deficit is reported in *mdx* mice only at the late stage of life, with a decline in hemodynamic parameters (35). To examine the cardiac function of the old *mdx* cohort, we isolated hearts and performed ex vivo cardiac perfusion using Langendorff chambers. Under high workload conditions induced by high calcium concentration, the *mdx*^4cv^ group treated with saline showed a clear deficit of the rate pressure product and positive maximum rate of change of derivative of pressure/derivative of pressure, while in the text (dP/dT_(max)_), and development of left ventricular pressure compared with WT age-matched littermates (Figure 7, A and B and Supplemental Figure 4). In contrast, hearts from *mdx*^4cv^ mice treated with the triple vector showed an effective response to high workload, with significant improvement of these hemodynamic parameters to WT levels. This response was the result of high protein levels of full-length dystrophin expressed from triple vectors in cardiac muscle, as found with Western blotting (Figure 7C). Immunostaining also revealed widespread dystrophin expression in these 24-month-old hearts, quantified in 40% of cardiomyocytes (Figure 7, D and E, and Supplemental Figure 5). Moreover, the analysis of heart cross-sections from the saline-treated group revealed the presence of scar tissue composed of collagen and infiltrated cells, as shown with H&E and trichrome staining (Figure 7, D and F). The extent of this scar tissue was greatly reduced in samples from the group treated with the triple AAV vectors (Figure 7, D and F).

These data show that the combination of low doses of myotropic AAVs and efficient split inteins led to the expression of therapeutic levels of full-length dystrophin in both skeletal and heart tissues when administrated to old and severely affected *mdx*^4cv^ mice, resulting in substantial functional improvements of the dystrophic phenotype.

## Discussion

Dystrophin is one of the larger proteins expressed by human cells and is involved in the pathogenesis of one of the most common genetic disorders (36). Its molecular organization in distinct domains with repetitive structures led to the generation of several truncated mini-proteins known as μDys that are currently being assessed in human clinical trials (7). Recent data from these trials suggest incomplete functional rescue of pathology in boys with DMD, which points to the necessity of inducing the expression of larger dystrophins (37). Several attempts have been made to restore expression of the full-length muscle isoform of dystrophin, but to date, obtaining therapeutic levels that rescue the DMD phenotype remains elusive. Our results showed efficient delivery and expression of full-length dystrophin in young and old *mdx*^4cv^ mice. Using low doses of the potent myotropic AAVMYO1 (38), broad biodistribution and high dystrophin expression were achieved, resulting in functional improvements of skeletal muscles and hearts and, to a lesser extent, diaphragm muscles.

Our data demonstrate remarkable correction of DMD muscle defects when the triple vectors were administrated at an early stage of the disease, with normalization of muscle force of both the hind limb and diaphragm muscles. However, at the late stage of the disease, the triple vector strategy corrected defects in hind limb muscles and the cardiac deficit but had a minimal effect on the diaphragm muscle. At this advanced phase of the disease, fewer myofibers remain in the diaphragm muscles, and important tissue remodeling was noticed with high collagen content and mononuclear cell infiltration, which likely explains the lack of therapeutic efficacy in this muscle. Additional studies are required to assess the long-term bioavailability of full-length dystrophin expressed with this strategy and whether early intervention would protect the diaphragm decline seen in aging *mdx*^4cv^ mice.

The results also revealed differing effects on TA myofiber central nucleation. In the younger cohort, robust expression of full-length dystrophin was detected with both doses in 40%–60% of myofibers, yet the percentage of myofibers with central nucleation remained above 60% twelve weeks after infusion (Figure 2C). These data align with our previous study, in which high levels of μDys5 or midi-Dys (ΔSR5-15) were expressed following systemic delivery of AAVMYO1 to 8-week-old *mdx*^4cv^ mice, where a high percentage of myofibers with central nuclei were also quantified. In both cases, muscles from the 8-week-old mice had already undergone a degeneration/regeneration crisis, which results in high numbers of centrally nucleated myofibers prior to vector administration (39). An earlier study of ours showed that administering AAV-μDys to young adult *mdx* mouse muscles led to a slow decline of central nucleation that was still incomplete 5 months after injection, however (7). Also, AAV-μDys injection into neonatal *mdx*^4cv^ mice prevented the development of central nucleation (9). In contrast, the expression of full-length dystrophin in older animals for a longer period (7 months) showed a significant reduction of centrally nucleated fibers compared with shorter time points in younger mice (Figure 6E). Together, these results suggest that earlier intervention and longer expression intervals may both be needed for maximal effect, but additional studies are needed for clarity (10, 40).

Moreover, in this study, the triple AAVs were delivered at an equimolar ratio of [1:1:1] using either a low dose (4 × 10^13^ total vg/kg) or a medium dose (8 × 10^13^ total vg/kg). Because each fragment has distinct stability and half-life, adjustment of the stoichiometry and/or selection of different expression cassettes with variable transcription activity should lead to higher levels of the final therapeutic product (i.e., full-length dystrophin), while limiting the accumulation of individual components (i.e., N-terminal [N-ter], middle, or C-terminal [C-ter] fragments, Supplemental Figure 6), as suggested by our previous work and others (23, 41). Nonetheless, developing sensitive assays to detect subtle changes in protein expression, such as ELISA or mass spectrometry, is a prerequisite for identifying optimal conditions that drive the highest protein expression.

The 2 split inteins used in this study have been optimized for minimal amino acid footprint scar following the intein splicing (23). To minimize the effect of the footprint on the dystrophin structure, split inteins were rationally inserted into loops between helices A and B of spectrin repeat 8 or within hinge 3. Our in vivo data support the idea that these modifications were well tolerated, as reflected by the long-term expression and functional amelioration of dystrophic defects found in both young and old mouse cohorts. Nonetheless, many other potential splitting sites clearly exist. Whether these changes to the protein sequence create a new epitope that elicits an immune response remains unclear. The identification or the development of new split intein pairs with promiscuous activity driving traceless PTS is suitable for enhancing the safety of this strategy. Similarly, most split intein sequences are of microbial origin, and their expression and accumulation in human cells may trigger an immune reaction, which would jeopardize this therapeutic approach. The intracellular half-life and fate of most split inteins in eukaryote cells are unknown. The development of specific antibodies or methods to detect their expression and localization is important before implementation in the clinic.

Furthermore, our study used the first generation of myotropic AAVMYO1 (38). Several new myotropic vectors have been engineered and are being evaluated in animal models of musculoskeletal diseases (42–44). While many of these new serotypes are showing high muscle tropism at very low doses with liver-detargeted biodistribution, the identification of a capsid with the same activity in human clinical trials poses the next challenge. Also, most of these newly engineered capsids are derived from natural serotypes. Many patients have been exposed to natural capsids, and whether the antibodies developed from the first exposure would recognize these new myotropic capsids remains unclear.

Finally, using dual AAV injections, PTS mediated by single split inteins has been successfully validated for mid-size proteins such as ABCA4 and factor F8, respectively, in Stargardt disease and hemophilia animal models (26, 28). Our results demonstrate the feasibility of expressing a therapeutic protein from multiple fragments deliverable by AAV vectors, which could be relevant to many genetic diseases caused by loss-of-function mutations in extra-large genes, such as nemaline- or RYR1-related myopathies. Nevertheless, the development of multi-AAV vector therapies could be challenging for human applications. Although the cost of such therapies can be reduced by the administration of low doses of potent and effective myotropic vectors, the regulatory aspect of approving these new classes of therapeutics may constitute a major hurdle.

## Methods

### Sex as a biological variant.

*mdx*^4cv^ female and male mice were used for breeding and generation of the mouse cohorts used in this study, but because DMD is an X-linked disease, thus only male mice were studied.

### Animals.

Dystrophin-deficient *mdx*^4cv^ or age-matched WT (C57BL/6) male mice were used in this study. Mice were randomized to experimental groups and assigned a serial identification number to conduct a blinded study. These numbers were used throughout the study, and the treatment history of each mouse was determined after completing the data collection.

### Plasmid subcloning and AAV production.

The split inteins/dystrophin constructs were previously cloned and validated in vitro using human embryonic kidney 293 (HEK 293) cells (23). Briefly, several split inteins were screened for their PTS activity to reassemble a reporter protein (GFP). The best pairs were then tested for their orthogonality. Two combinations of split inteins with highly specific activity were selected and subcloned into a pAAV plasmid containing human dystrophin sequences, a small synthetic polyA signal, and the muscle-specific M-creatine kinase (CK) 8e expression cassette (CK8e). AAVMYO1 vectors carrying split intein/dystrophin N-ter, middle, or C-ter sequences were generated using the triple plasmid transfection method of HEK293 cells as previously described (45). The HEK293 subclone was a gift from the Dusty Miller laboratory (Fred Hutch Cancer Center, Seattle, Washington, USA) to the Virus Production Core of the Wellstone Research Center (University of Washington, Seattle, Washington, USA), which was authenticated and attested to be negative for *Mycoplasma* by Bionique Testing Laboratories Inc.

### AAV administration.

Mice were anesthetized using isoflurane (Piramal Critical Care). A total dose of 4 × 10^13^ or 8 × 10^13^ vg/kg at a ratio of [1:1:1] of split Nter, middle, C-ter intein/dystrophin vectors was administrated systemically into the circulation using the tail vein route in 8-week-old or 17-month-old mice. Sterile saline solutions were injected as a sham manipulation. Once AAV or saline solutions were successfully administrated, mice were kept in a warm cage and monitored for 1 hour.

### TA and diaphragm contractile properties.

The muscle force–generating capacity of young and old mice was assessed at 3 and 7 months after AAV infusion, respectively. TA muscle–specific force and susceptibility to contraction-induced injury were determined in situ after sciatic nerve stimulation (Aurora Scientific, model 701C), and the diaphragm maximal force was measured in vitro on longitudinal and intact muscle strips freshly isolated from young or old mice using a temperature-controlled in vitro horizontal bath (Aurora Scientific, model 809A) filled with carbogen-bubbling Tyrode’s solution. Briefly, the maximum isometric tetanic force was determined at optimal muscle length (L0) following 200 Hz (TA) or 180 Hz (diaphragm strips) stimulations (see Supplemental Tables 1 and 2). TA muscle eccentric contractions were performed by stimulating the muscle at the fixed and predetermined L0, allowing peak isometric force to be generated for 150 ms (TA). While the muscle remained stimulated for an additional 200 ms, the muscle was subjected to a series of physical lengthenings of 5%, 10%, 15%, or 20% with 30-second intervals of rest between each measurement. The response from an eccentric contraction was measured by the peak isometric force generated just prior to the subsequent eccentric contraction. The obtained values were then compared with the initial measurement at L0 and presented as a the Δ force drop.

### Ex vivo cardiac function.

Hearts were isolated from deeply anesthetized old mice and quickly perfused with modified Krebs-Henseleit buffer supplemented with glucose and pyruvate (118 mM NaCl, 25 mM NaHCO_3_, 5.3 mM KCl, 2.0 mM CaCl_2_, 1.2 mM MgSO_4_, 0.5 mM ethylenediaminetetraacetic acid, 10.0 mM glucose, and 0.5 mM pyruvate) and equilibrated with 95% O_2_ and 5% CO_2_ at pH 7.4. Using the Langendorff chamber, hearts were perfused at a constant pressure of 80 mmHg and maintained at 37.5°C throughout the protocol. After 5 minutes of stabilization, the perfusate was changed to an buffer identical to the one described above, except for the addition of 4.0 mM CaCl_2_ to simulate a high-workload challenge for 20 minutes. The pressure developed in the left ventricle, the heart rate, and the minimum and maximum rates of pressure change in the ventricle (±dP/dt) were measured at the baseline or under high-workload conditions by inserting a water-filled balloon into the left ventricle, which was connected to a pressure transducer (PowerLab, ADInstruments). The left ventricle balloon volume was adjusted during the equilibration period to achieve an end-diastolic pressure of 8–10 mmHg and was unchanged for the duration of the experiment.

### Muscle histology analysis.

TA, diaphragm, and heart muscles were collected and snap-frozen in liquid nitrogen–cooled isopentane, followed by cryosectioning into 10 μm cross-sections using a cryostat (Leica, CM1850), and later stained with H&E or trichrome. Whole sections were imaged with the Hamamatsu NanoZoomer slide scanner. Other sections were immunolabeled with antibodies against dystrophin N-terminal (homemade rabbit 246), γ-sarcoglycan (NCL-g-SARC, Leica Biosystems), β-dystroglycan (NCL-b-DG, Leica Biosystems), or laminin 2 (L0663 rat, MilliporeSigma). The secondary antibodies used were goat anti–rabbit Alexa-Fluor 350 (A-21068, Invitrogen, Thermo Fisher Scientific), goat anti–rabbit Alexa-Fluor 488 mouse IgG2a (Jackson ImmunoResearch Laboratories, 115-547-186), goat anti–rabbit Alexa-Fluor 594 mouse IgG2b (Jackson ImmunoResearch Laboratories, 115-587-187), or goat anti-rat Alexa-Fluor 680 (Jackson ImmunoResearch 712-625-150). Slides were mounted using Immu-Mount (Epredia), and images were captured on a Nikon Eclipse 90i microscope. The percentage of myofibers with centralized nuclei, the myofiber size, the minimal fiber diameter (miniFeret), and the fibrosis area were measured using Fiji image analysis software (version 2.0.0-rc-68/1.52g).

### Protein extraction and expression analysis.

Total proteins were extracted using RIPA supplemented with 1 mM PMSF and a 5% protease inhibitor cocktail (P8340, MilliporeSigma). Then, the protein concentration was determined using the Pierce BCA assay kit (Thermo Fisher Scientific). Samples were denatured at 100°C for 10 minutes, and then 30 μg protein lysates were separated in NuPage 4%–12% Bis-Tris polyacrylamide gels (Invitrogen, Thermo Fisher Scientific). Protein transfer onto 0.45 μm PVDF membranes (Amersham hybond) was performed at 120 V at 4°C for 2 hours. Membranes were blocked for 2 hours in Tris-buffered saline containing 5% nonfat dry milk and 0.005% Tween 20 before overnight incubation with antibodies against dystrophin N-terminal (mouse Manex1011b, DSHB), middle (Leica Dys1), C-terminal (Leica Dys2), or GAPDH (rabbit, MilliporeSigma G9545) as a loading control. The secondary antibodies coupled to HRP were: anti–mouse IgG2a (Jackson ImmunoResearch Laboratories, 115-035-206), anti–mouse IgG1 (Jackson ImmunoResearch Laboratories, 115-035-205), or goat anti-rabbit (Jackson ImmunoResearch Laboratories, 111-035-144). Blots were incubated for 2 hours at room temperature before visualization using Clarity Western ECL substrate (Bio-Rad) in the Chemidoc MP imaging system (Bio-Rad). Relative expression was determined by band densitometric measurements on unsaturated images using Fiji image analysis software.

### Statistics.

All data are organized by groups using Microsoft Excel, version 16.72. Graphs were made using GraphPad Prism, version 10.2.0 335 (GraphPad Software). Values are expressed as the mean ± SEM with individual and biologically independent data points shown as dots, except for myofiber area and minimal Feret’s diameter, where the data distribution is presented in violin bars with median and quartile values. Significant differences were determined using 1-way ANOVA followed by Tukey’s test to determine the statistical significance among the groups. *P* values of less than 0.05 were considered statistically significant.

### Study approval.

All animal experiments were conducted following the animal care and use protocol approved by the IACUC of the University of Washington.

### Data availability.

All relevant data that support the findings of this study are available in the Supporting Data Values file or from the corresponding author upon reasonable request.

## Author contributions

HT and JSC designed and conceptualized the study. TSM performed the ex vivo heart perfusions. TRR collected muscle and non-muscle samples and prepared reagents, plasmids, and mice. RT and MR provided reagents and equipment for analysis of cardiac function. CLH titered the AAV preparations. HT produced and purified AAVs, carried out all other experiments, and wrote the manuscript with input from all co-authors. JSC provided reagents and advice and edited the manuscript.

## Supplementary Material

Supplemental data

Unedited blot and gel images

Supporting data values

## Version 1 06/10/2025

In-Press Preview

## Version 2 08/01/2025

Electronic publication
