# Supplementary material for: Expression of full-length dystrophin reverses muscular dystrophy defects in young and old mdx4cv mice
Source: J Clin Invest. 2025 Jun 10;135(15):e189075. doi: 10.1172/JCI189075 (PMC12321383; doi:10.1172/JCI189075)
Supplement: Supplemental data [file jci-135-189075-s192.pdf]

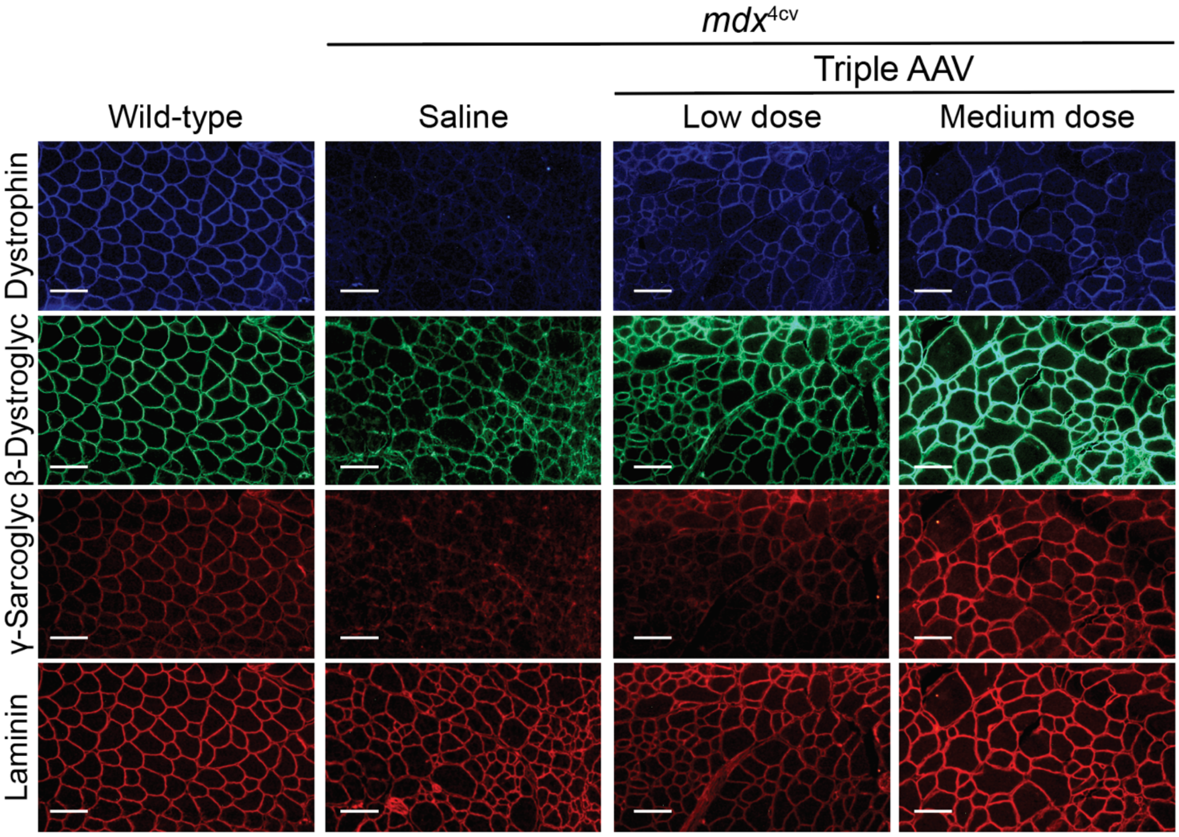

Supplementary Figure 1. The original panel presented in Figure 2a.

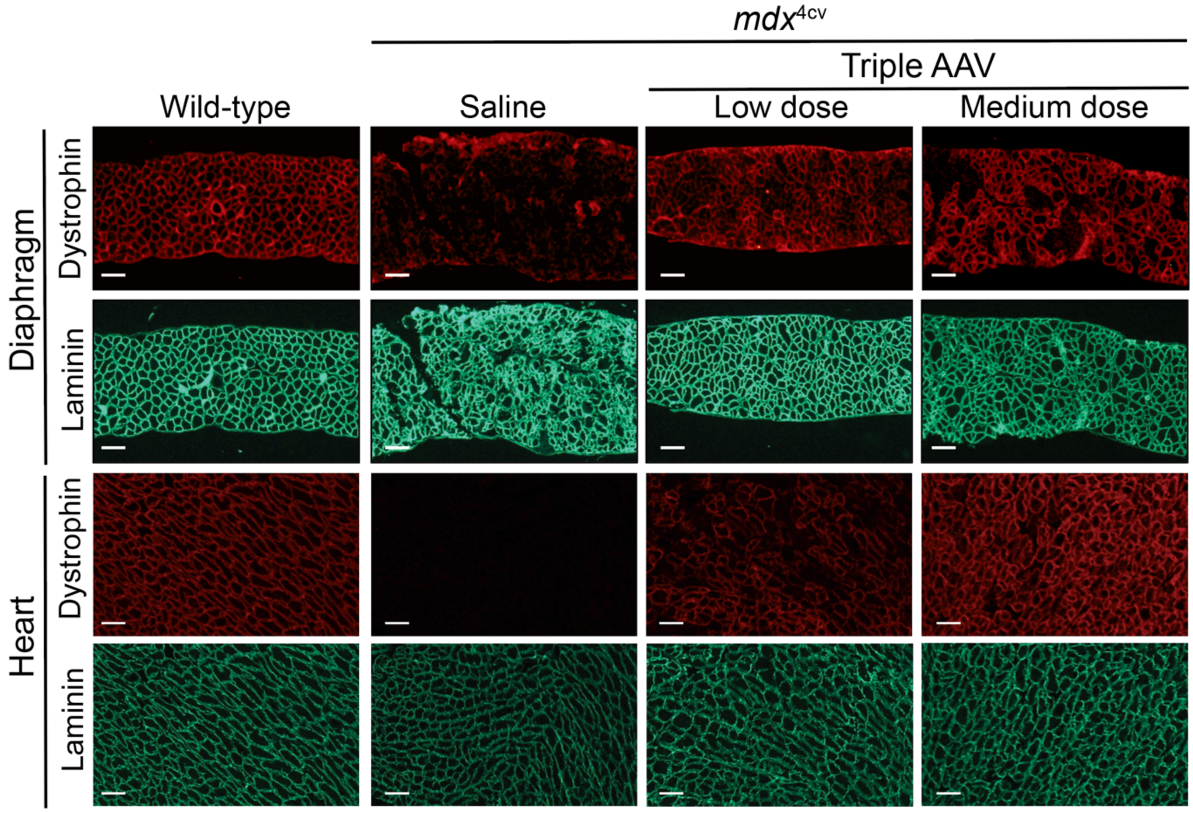

Supplementary Figure 2. The original immunostaining presented in Figure 3a.

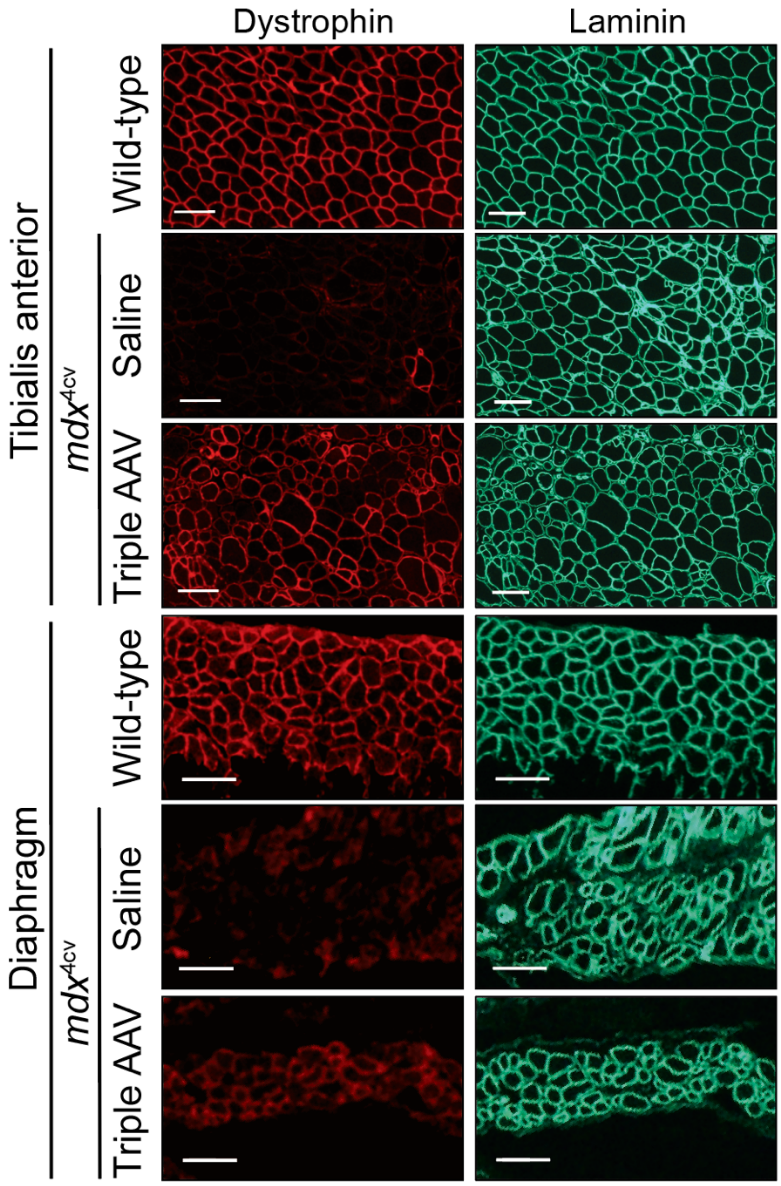

Supplementary Figure 3. The original immunostaining presented in Figure 6a.

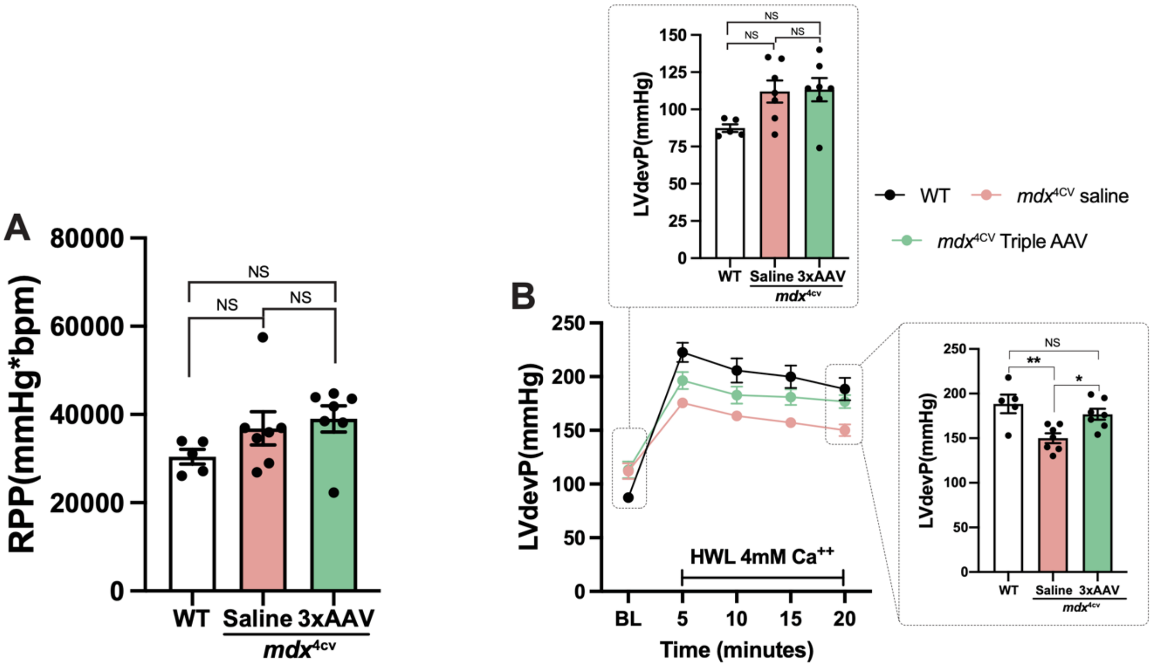

Supplementary Figure 4. A) Rate pressure product (RPP) at baseline (before the high workload challenge). **B)** Left developed pressure measured at baseline (BL) or at different time points under high workload conditions (HWL). N.S: Not statistically significant. **P<0.01 using ANOVA test followed by Tukey's *post hoc*.

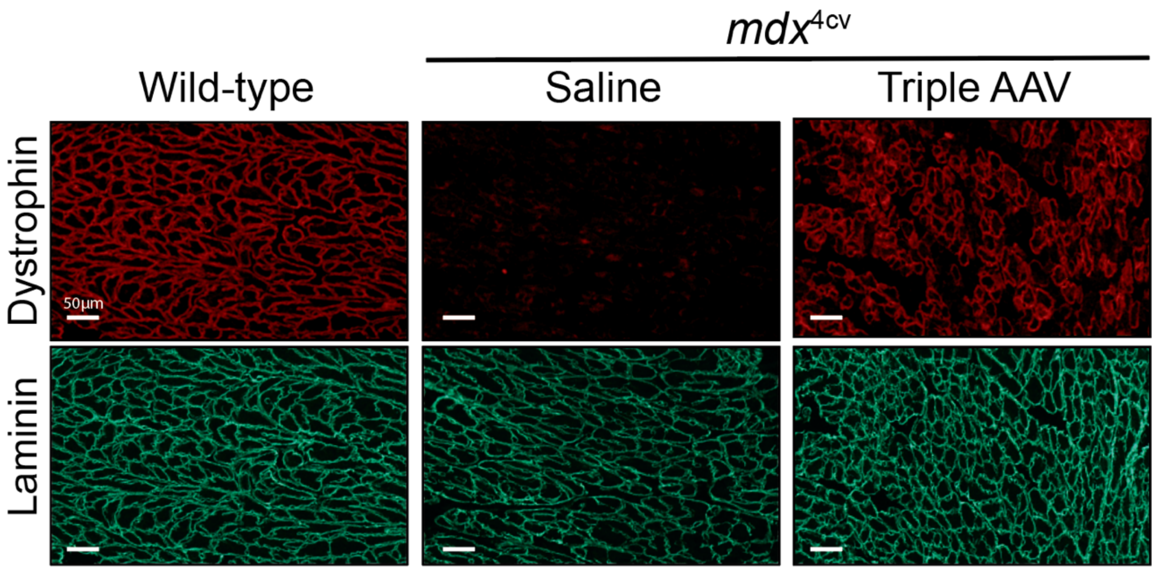

Supplementary Figure 5. The original panel presented in Fig 7a.

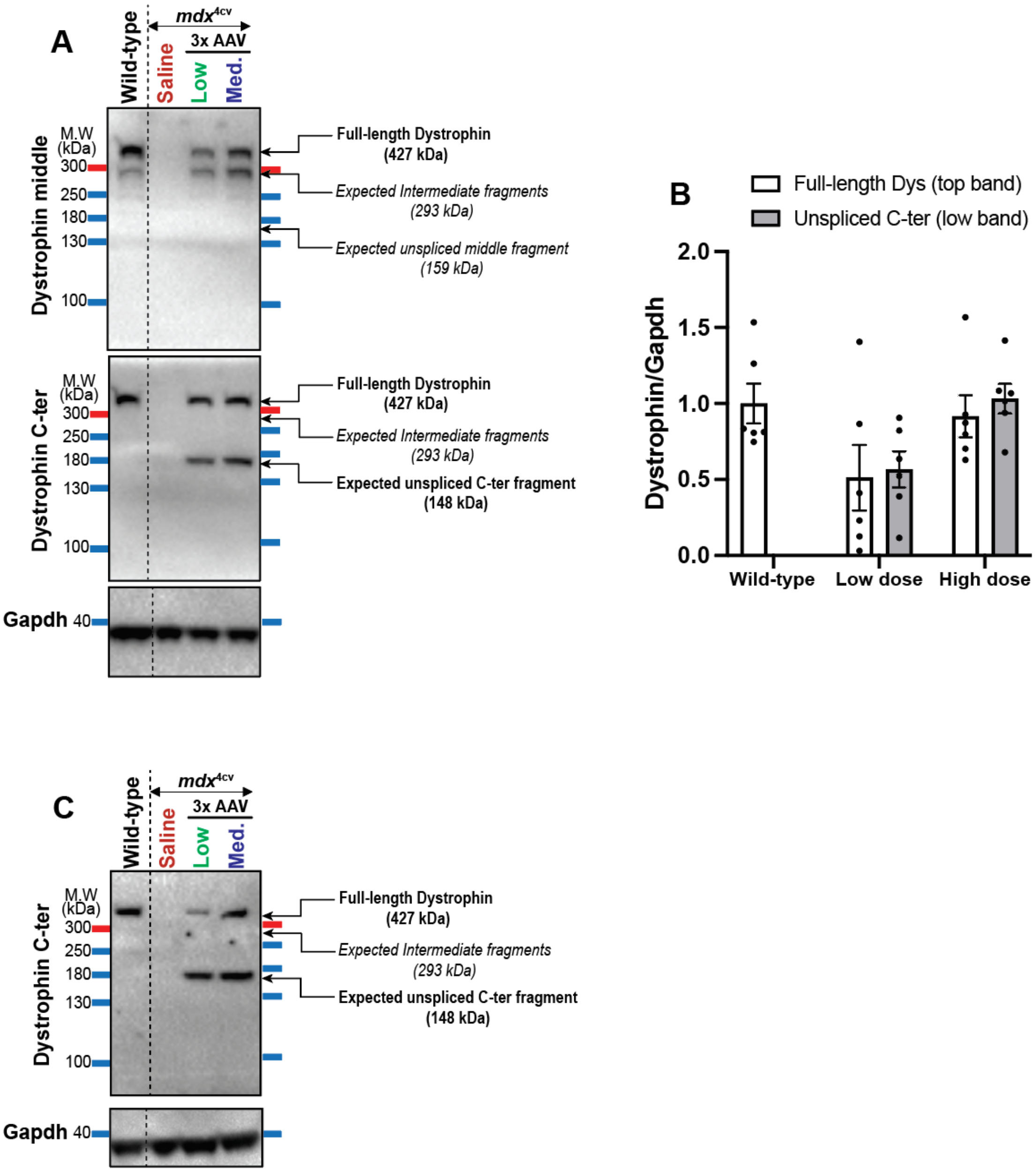

Supplementary Figure 6. Full-length dystrophin and partial fragments expression in TA muscles.

A) Protein expression detection using western blot probed with a specific antibody to either middle or C-terminal fragment. **B)** Densitometry quantification using two antibodies against the middle or C-terminal fragments of dystrophin. *n*=5 samples used. **C**) Western blot using protein lysates from heart samples probed with a specific antibody to the C-terminal fragment.

Supplementary Table 1: Absolute force, muscle mass and optimal L0 of TA muscles of young WT or *mdx*^{4cv} mice

	Wild-type					*mdx*4cv														
						Saline					Low dose triple AAVYO1					Medium dose triple AAVYO1				
	Absolute force (mN)	Muscle mass (mg)	L0 (mm)	CSA (mm2)	Specific force (kN/m2)	Absolute force (mN)	Muscle mass (mg)	L0 (mm)	CSA (mm2)	Specific force (kN/m2)	Absolute force (mN)	Muscle mass (mg)	L0 (mm)	CSA (mm2)	Specific force (kN/m2)	Absolute force (mN)	Muscle mass (mg)	L0 (mm)	CSA (mm2)	Specific force (kN/m2)
**Mouse 1**	1406	59.7	14	6.7	209.7	1246	82.7	14	9.3	134.2	1449	88.4	14	9.9	145.9	1206	59.7	14	6.7	179.9
**Mouse 2**	1169	60.8	15	6.4	183.4	1283	79.6	14	8.9	143.5	1990	103.8	14	11.7	170.7	1306	79.7	14	9.0	145.9
**Mouse 3**	1124	58.1	14	6.5	172.3	1192	124.2	15	13.0	91.6	950	71.2	14	8.0	118.8	1791	93	15	9.7	183.7
**Mouse 4**	1256	56	14	6.3	199.7	1085	70.5	14	7.9	137.0	1477	79	14	8.9	166.5	1378	60.1	14	6.7	204.2
**Mouse 5**	1652	57.8	14	6.5	254.5	937	69.8	14	7.8	119.5	1302	60.4	14	6.8	191.9	1289	76	15	8.0	161.8
**Mouse 6**	1242	55	14	6.2	201.1	1119	130.3	15	13.7	81.9	1805	99.1	14	11.1	162.2	1279	78.2	15	8.2	156.0
**Mouse 7**	1185	50.5	14	5.7	208.9	1539	107.5	14	12.1	127.5	/	/	/	/	/	1812	79.8	15	8.4	216.6
**Average**	**1290.6**	**56.8**	**14.1**	**6.3**	**204.2**	**1200.1**	**94.9**	**14.3**	**10.4**	**119.3**	**1495.5**	**83.7**	**14.0**	**9.4**	**159.3**	**1437.3**	**75.2**	**14.6**	**8.1**	**178.3**
**SEM**	**69.2**	**1.3**	**0.1**	**0.1**	**9.8**	**71.2**	**9.6**	**0.2**	**0.9**	**8.9**	**150.3**	**6.8**	**0.0**	**0.8**	**10.1**	**96.0**	**4.5**	**0.2**	**0.4**	**9.7**

Supplementary Table 2: Absolute force, muscle mass and optimal L0 of diaphragm strips isolated from young WT or *mdx*^{4cv} mice

	Wild-type					*mdx*4cv														
						Saline					Low dose triple AAVYO1					Medium dose triple AAVYO1				
	Absolute force (mN)	Muscle mass (mg)	L0 (mm)	CSA (mm2)	Specific force (kN/m2)	Absolute force (mN)	Muscle mass (mg)	L0 (mm)	CSA (mm2)	Specific force (kN/m2)	Absolute force (mN)	Muscle mass (mg)	L0 (mm)	CSA (mm2)	Specific force (kN/m2)	Absolute force (mN)	Muscle mass (mg)	L0 (mm)	CSA (mm2)	Specific force (kN/m2)
**Mouse 1**	61.5	4	9	0.41	146.7	32.88	8.8	9	0.922	35.6	106.24	8	9	0.83	126.7	82.88	5.2	9	0.54	152.1
**Mouse 2**	262	8.5	9	0.89	294.1	58.2	12.1	10	1.14	51.0	76.18	5.3	9	0.55	137.1	63.18	7.7	10	0.72	87.0
**Mouse 3**	88.84	4.1	9	0.42	206.7	106.38	9.9	9	1.03	102.5	115.14	9	9	0.94	122.0	114	7.8	9	0.81	139.4
**Mouse 4**	215.26	8	9	0.83	256.7	30.62	6.8	9	0.71	43.0	178.2	7.9	9	0.82	215.2	103.12	6.5	9	0.68	151.3
**Mouse 5**	223.26	8	9	0.83	266.2	53.7	7.3	8	0.86	62.4	97.48	5.5	9	0.57	169.1	94.24	5.9	9	0.61	152.4
**Mouse 6**	140.36	8.6	9	0.90	155.7	23.46	12.7	9	1.33	17.6	96.18	9.2	9	0.96	99.7	79.39	5.3	10	0.5	158.8
**Average**	**165.2**	**6.9**	**9.0**	**0.7**	**221.0**	**50.9**	**9.6**	**9.0**	**1.0**	**52.0**	**111.6**	**7.5**	**9.0**	**0.8**	**145.0**	**89.5**	**6.4**	**9.3**	**0.6**	**140.2**
**SEM**	**32.9**	**0.9**	**0.0**	**0.1**	**24.9**	**12.4**	**1.0**	**0.3**	**0.1**	**11.8**	**14.3**	**0.7**	**0.0**	**0.1**	**16.8**	**7.4**	**0.5**	**0.2**	**0.0**	**10.9**
