# Supplementary material for: Expression of full-length dystrophin reverses muscular dystrophy defects in young and old mdx4cv mice
Source: J Clin Invest. 2025 Jun 10;135(15):e189075. doi: 10.1172/JCI189075 (PMC12321383; doi:10.1172/JCI189075)

Figure 1C (Tibialis anterior)

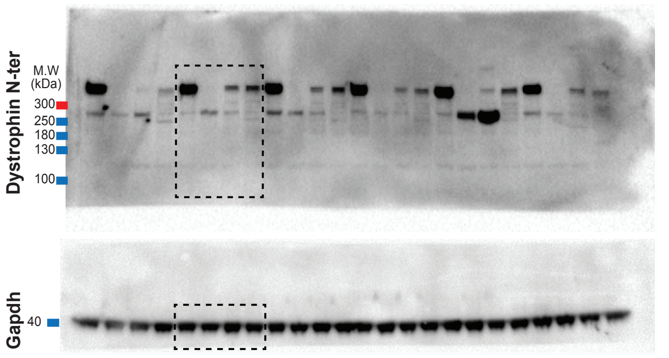

Figure 1C (Heart)

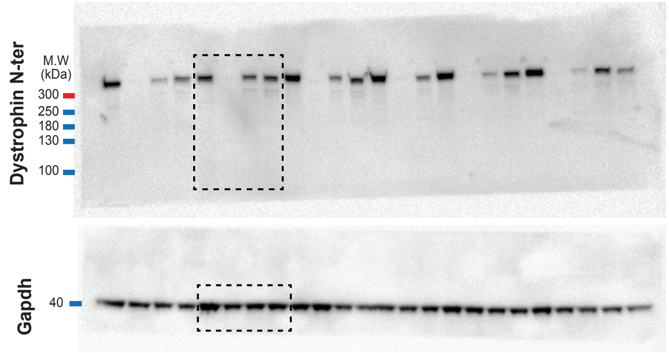

Figure 1C (diaphragm)

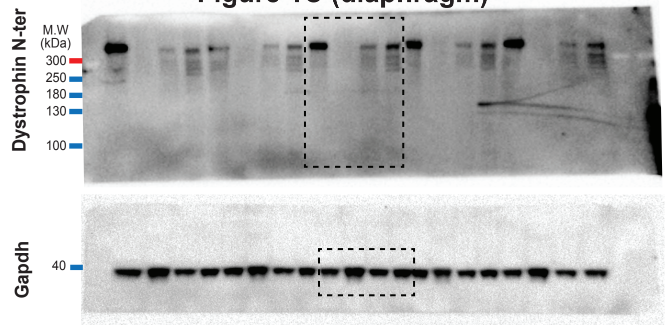

Supplementary Figure 6A

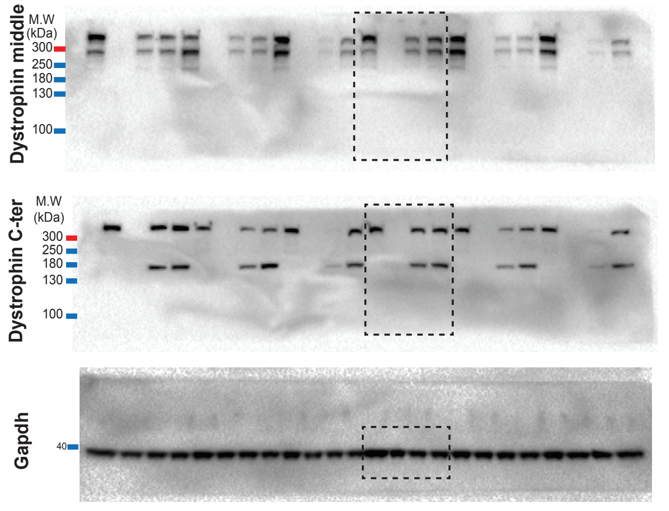

Figure 5D (Tibialis anterior)

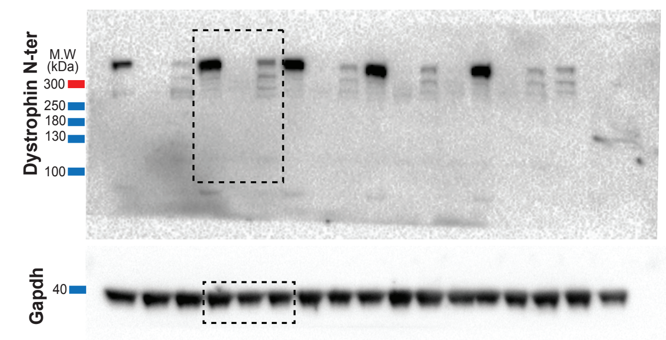

Figure 5D (diaphragm)

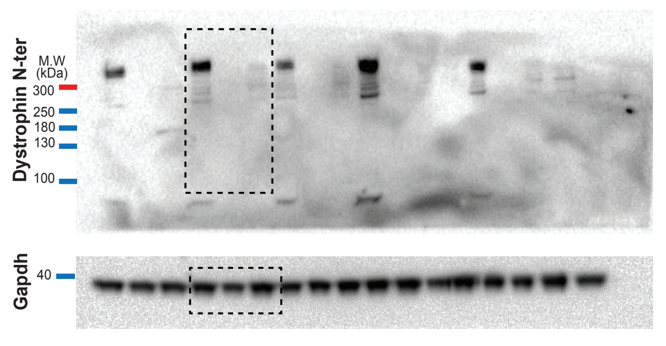

Figure 7C (Heart)

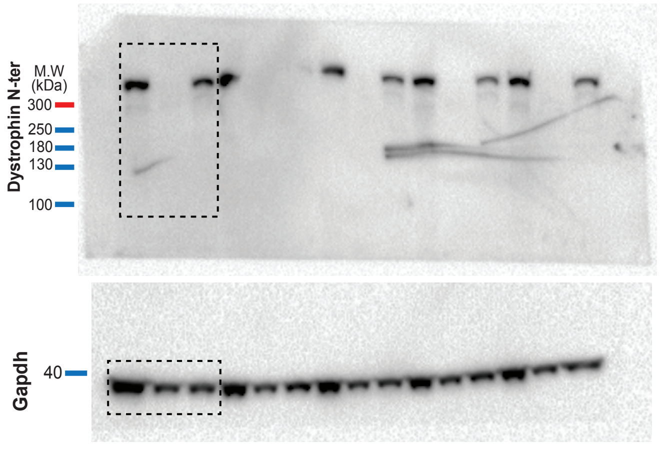

Supplement: Unedited blot and gel images [file jci-135-189075-s193.pdf]
